# Interstitial changes after reperfused myocardial infarction in swine: morphometric and genetic analysis

**DOI:** 10.1186/s12917-020-02465-6

**Published:** 2020-07-29

**Authors:** Cesar Rios-Navarro, Maria Ortega, Victor Marcos-Garces, Jose Gavara, Elena de Dios, Nerea Perez-Sole, Francisco J. Chorro, Vicente Bodi, Amparo Ruiz-Sauri

**Affiliations:** 1 INCLIVA Health Research Institute, Valencia, Spain; 2 Pathology Department, School of Medicine, University of Valencia, Av Blasco Ibañez 15, 46010 Valencia, Spain; 3 Cardiology Department, Hospital Clinico Universitario, Av Blasco Ibanez, 17 46010 Valencia, Spain; 4 Department of Medicine, School of Medicine, University of Valencia, Valencia, Spain; 5 Centro de Investigación Biomédica en Red – Cardiovascular (CIBER-CV), Madrid, Spain

**Keywords:** Extracellular matrix, Myocardial infarction, Swine

## Abstract

**Background:**

Following myocardial infarction (MI), we aimed to characterize morphometric and genetic changes in extracellular matrix (ECM) components from ischemia onset until late phases after coronary reperfusion in necrotic and salvaged myocardium.

**Results:**

Swine were divided into one control (*n* = 5) and three MI groups: 90-min of ischemia without reperfusion, or followed by 1-week or 1-month reperfusion (*n* = 5 per group). In samples from the necrotic and salvaged areas, ECM components were morphometrically quantified and mRNA levels of factors involved in ECM remodeling were evaluated. After 90-min of ischemia, fibronectin, laminin, and elastic fibers content as well as upregulated mRNA expression of tissue inhibitors of metalloproteinases (TIMP)1, TIMP2, TIMP3 and connective tissue growth factor increased in the necrotic and salvaged myocardium. In both reperfused MI groups, collagen-I, collagen-III, elastic fibers, glycosaminoglycans, laminin, and fibronectin levels heightened in the necrotic but not the salvaged myocardium. Moreover, mRNA expression of TIMP1, TIMP2 and TIMP3, as well as metalloproteinase-2 and metalloproteinase-9 heightened in the necrotic but not in the salvaged myocardium.

**Conclusions:**

Matrix remodeling starts after ischemia onset in both necrotic and salvaged myocardium. Even if ECM composition from the salvaged myocardium was altered after severe ischemia, ECM makes a full recovery to normal composition after reperfusion. Therefore, rapid coronary reperfusion is essential not only to save cardiomyocytes but also to preserve matrix, thus avoiding impaired left ventricular remodeling.

## Background

The extracellular matrix (ECM) is an imbricated network made up of fibers and ground substance (composed mainly of glycosaminoglycans (GAG), proteoglycans and adhesion glycoproteins). It provides structural support for different cell types, modifies cellular activity and actively participates in regulating survival, migration, proliferation, apoptosis, and inflammatory responses [1–3].

Myocardial infarction (MI) is caused by the thrombotic occlusion of a coronary artery. Current gold-standard therapy for ST-segment elevation MI patients is prompt coronary revascularization to reestablish nutrient and oxygen supply in the downstream myocardium [4]. Although percutaneous intervention has played a pivotal role in saving viable cardiomyocytes as well as reducing infarct size and cardiac events [5–7], heart failure nonetheless occurs in post-MI patients, mainly due to adverse ventricular geometry and wall thinning [8, 9], and to prevent this, localized and timely regulated ECM remodeling is mandatory after MI [3, 10, 11].

Despite the role of ECM after MI has been previously reported [2, 12], we aim to comprehensively examine the morphometric and molecular changes in the ECM occurring in the necrotic and salvaged myocardium from ischemia onset until late phase post-reperfusion. In a swine model of reperfused MI, the specific objectives of this study are: 1) to use morphometric analysis to quantify the presence and organization of ECM components throughout the ischemia-reperfusion process following MI, 2) to compare ECM changes between necrotic and salvaged myocardium, and 3) to pinpoint the dynamics of key genes responsible for ECM remodeling in the necrotic and salvaged myocardium.

## Results

Experiments were successfully performed in controls (*n* = 5). Ninety-min mid-LAD coronary artery occlusion was conducted in 20 pigs, three of them died during balloon inflation due to refractory ventricular fibrillation and two during the reperfusion period. Experiments were successfully completed in the remaining 15 cases, although electrical ventricular defibrillation was needed during coronary occlusion or immediately after reperfusion in 4 cases. Patency of the LAD was confirmed in all cases before sacrifice. Five controls and 15 MI experiments (without coronary reperfusion or followed by 1-week or 1-month reperfusion; *n* = 5 animals in each experimental group) made up the final study group. A sample size of 5 swine in each experimental was based on previous literature not only from our group [13, 14] and others [15–17].

### Morphometric changes in the ECM composition after 90-min of ischemia without coronary reperfusion

Even though left ventricular geometry after 90-min of ischemia was similar to control hearts, some fluctuations in ECM composition were detected (Table 1).

**Table 1:** Macroscopic evaluation of LV geometry

	CONTROL	ISCHEMIA WITHOUT REPERFUSION	1-WEEK REPERFUSION	1-MONTH REPERFUSION
LAD-perfused area (% of LV)		64 ± 6	76 ± 8	66 ± 13
Infarct area (% LAD perfused area)		0 ± 0	34 ± 13**	26 ± 11**
Myocardial wall thickness, mm				
Necrotic area	10 ± 2.3	10 ± 2.0	9 ± 2.7	6 ± 2.9*^#^
Salvaged area	10 ± 2.3	11 ± 2.1	11 ± 2.5	11 ± 3.8

Data were expressed as mean ± SD (*n* ≥ 5) and were analysed by unpaired t-Student’s test. *P < 0.05, **P < 0.01 vs. control; ^#^*P* < 0.05 vs. corresponding salvaged area

Abbreviations: *LAD* Left anterior descending; *LV* Left ventricle

**Fig. 1:**
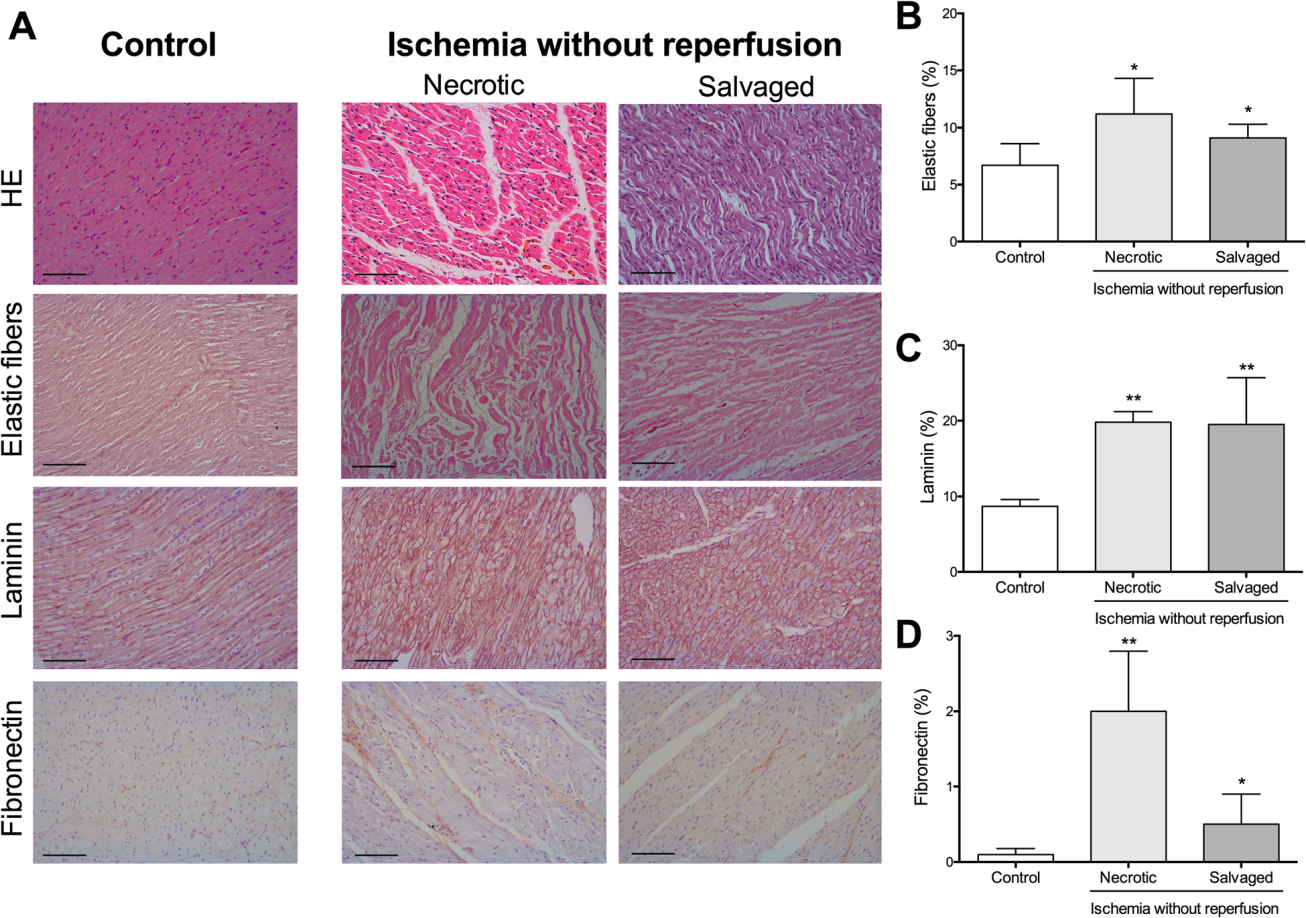
Morphometric analysis of extracellular matrix components in the necrotic and salvaged myocardium isolated after 90-min of ischemia without reperfusion. (**a**) Representative images from control group (left panel) and the necrotic (central panel) and salvaged (right panel) myocardium isolated from the 90-min ischemia without reperfusion group stained with hematoxylin-eosin (HE) (upper panel) and orcein for elastic fibers (upper-middle panel) as well as with the specific marker for laminin (lower-middle panel), and fibronectin (lower panel). The scale bars indicate 50 μm. Morphometric quantification of elastic fibers (**b**), laminin (**c**), and fibronectin (**d**). Images were analyzed with Image-Pro Plus analysis software. The area occupied by elastic fibers, laminin, and fibronectin was increased in the necrotic and salvaged myocardium isolated from the no reperfusion group in comparison to the control myocardium. Data (mean ± SD, *n* ≥ 5) were analysed by non-paired t-Student’s test. Scoring was performed by a blinded observer unaware of the experimental group. *P < 0.05, **P < 0.01 vs. control

In the no reperfusion group group (Fig. 1a), an increased amount of elastic fibers was detected in the necrotic and salvaged myocardium compared to the control heart group (Fig. 1b), displaying a disorganized disposition among the cardiomyocytes. There was a similar amount (Table 2), disposition, evaluated using Fast Fourier transform analysis (Fig. S1), and mRNA expression (Table 3) of collagen type I and type III in necrotic and salvaged myocardium in comparison to control tissue.

**Table 2:** Values obtained from morphometric analysis of ECM components

	CONTROL	ISCHEMIA WITHOUT REPERFUSION		1-WEEK REPERFUSION		1-MONTH REPERFUSION	
		NECROTIC	SALVAGED	NECROTIC	SALVAGED	NECROTIC	SALVAGED
Collagen I (%)	20.3 ± 10.1	24.3 ± 6.7	18.9 ± 12.1	53.0 ± 10.1**	18.1 ± 12.0	67.1 ± 3.8**	23.0 ± 3.1
Collagen III (%)	16.0 ± 9.3	21.9 ± 8.5	21.6 ± 8.4	24.6 ± 8.5	14.5 ± 8.5	36.6 ± 14.6*	17.9 ± 7.7
Elastic fibers (%)	6.7 ± 1.9	11.2 ± 3.1*	9.1 ± 1.2*	20.3 ± 6.2**	13.6 ± 7.5	16.4 ± 3.0**	12.6 ± 7.4
GAG (%)	10.9 ± 7.1	5.9 ± 1.4	5.1 ± 1.3	29.2 ± 5.0**	16.4 ± 10.0	25.4 ± 8.3**	14.1 ± 6.3
SPARC (%)	2.5 ± 0.6	0.4 ± 0.4	2.1 ± 0.5	6.9 ± 2.0**	1.4 ± 0.9	6.6 ± 1.2**	1.2 ± 0.3
Perlecan (%)	0.7 ± 0.4	0.7 ± 0.2	0.9 ± 0.2	0.3 ± 0.2	0.1 ± 0.2	1.1 ± 0.7	0.4 ± 0.4
Laminin (%)	8.7 ± 0.9	19.8 ± 1.4**	19.5 ± 6.2**	14.3 ± 1.5**	11.5 ± 1.3*	17.7 ± 1.1**	5.5 ± 4.2
Fibronectin (%)	0.1 ± 0.1	2.0 ± 0.8**	0.5 ± 0.4*	13.3 ± 9.8*	0.6 ± 0.7	7.2 ± 4.1**	0.4 ± 0.6

Data were expressed as mean ± SD (*n* ≥ 5) and were analysed by unpaired t-Student’s test. *P < 0.05, **P < 0.01 vs. control

Abbreviations: *ECM* Extracellular matrix; *GAG* Glycosaminoglycan; *SPARC* Secreted protein acidic and rich in cysteine

**Table 3:** mRNA levels of factors involved in ECM remodeling

	CONTROL	ISCHEMIA WITHOUT REPERFUSION		1-WEEK REPERFUSION		1-MONTH REPERFUSION	
		NECROTIC	SALVAGED	NECROTIC	SALVAGED	NECROTIC	SALVAGED
TNF-α	1.10 ± 0.54	6.52 ± 4.44	6.26 ± 3.18	20.59 ± 11.10**	7.54 ± 6.17	60.98 ± 21.55**	11.30 ± 10.35
BSG	1.01 ± 0.18	1.35 ± 0.25	1.31 ± 0.22	0.38 ± 0.07*	0.27 ± 0.22*	0.74 ± 0.47	0.52 ± 0.50
TGF-β	1.06 ± 0.42	2.06 ± 0.24	1.81 ± 0.32	2.79 ± 1.49	0.48 ± 0.22	16.62 ± 12.04**	0.65 ± 0.79
CTGF	1.24 ± 0.77	7.40 ± 1.27**	3.01 ± 1.00*	3.10 ± 2.66	0.28 ± 0.09	28.25 ± 13.64**	0.98 ± 0.32
MMP2	1.05 ± 0.13	1.98 ± 0.65	3.26 ± 1.30	27.08 ± 7.5**	3.95 ± 2.28	15.97 ± 4.24**	4.09 ± 2.26
MMP9	1.02 ± 0.22	5.79 ± 7.64	4.62 ± 4.08	95.98 ± 69.45*	7.05 ± 6.65	272.31 ± 140.89**	5.95 ± 6.70
MMP14	1.03 ± 0.22	2.78 ± 0.93	2.18 ± 0.71**	2.95 ± 2.54	0.28 ± 0.22*	6.21 ± 2.79*	0.15 ± 0.07*
MMP15	1.03 ± 0.30	2.12 ± 1.02	1.82 ± 0.90	1.13 ± 1.07	0.41 ± .13	1.13 ± 1.07	0.62 ± 0.73
MMP16	1.05 ± 0.35	2.43 ± 1.14	2.04 ± 0.58	1.15 ± 0.68	0.58 ± 0.32	6.39 ± 2.70*	1.55 ± 0.87
TIMP1	1.02 ± 0.23	6.50 ± 2.92*	5.64 ± 3.19*	27.14 ± 17.37**	1.92 ± 1.32	30.92 ± 18.14**	1.15 ± 0.75
TIMP2	1.00 ± 0.08	3.08 ± 0.98**	2.60 ± 0.85**	4.19 ± 1.77*	0.99 ± 0.86	32.50 ± 20.41**	0.89 ± 0.47
TIMP3	0.96 ± 0.15	2.82 ± 1.45*	1.77 ± 0.47	1.06 ± 0.88	0.58 ± 0.29	4.83 ± 2.80*	0.81 ± 0.40
TIMP4	1.29 ± 0.35	1.45 ± 0.59	1.51 ± 0.30	1.32 ± 0.45	0.37 ± 0.16**	1.14 ± 0.60	0.36 ± 0.21**
COL1A1	1.02 ± 0.25	3.99 ± 2.30	2.65 ± 1.54	10.58 ± 4.81	2.37 ± 1.07	45.71 ± 29.07**	2.17 ± 2.15
COL3A1	1.05 ± 0.37	2.86 ± 1.83	1.87 ± 0.84	4.39 ± 2.00	1.17 ± 0.81	18.03 ± 12.52*	1.14 ± 0.62
FN1	1.03 ± 0.52	3.76 ± 2.35	2.31 ± 0.75	17.84 ± 8.55**	1.75 ± 1.24	106.10 ± 43.39**	2.20 ± 2.25
LAMB2	1.08 ± 0.32	2.07 ± 0.97	1.97 ± 0.64	25.84 ± 8.60**	2.15 ± 0.51	54.04 ± 23.06**	2.09 ± 0.87

Abbreviations: *BSG* Basigin; *COL* Collagen; *CTGF* Connective tissue growth factor; *ECM* Extracellular matrix; *FN* Fibronectin; *LAMB*: Laminin; *MMP* Metalloproteinases; *TIMP* Tissue inhibitors of metalloproteinases: *TGF* Transforming growth factor; *TNF* Tumor necrosis factor

Data were expressed as mean ± SD (*n* ≥ 5) and were analysed by one-way ANOVA followed by Bonferroni test. *P < 0.05, **P < 0.01 vs. control

**Fig. 2:**
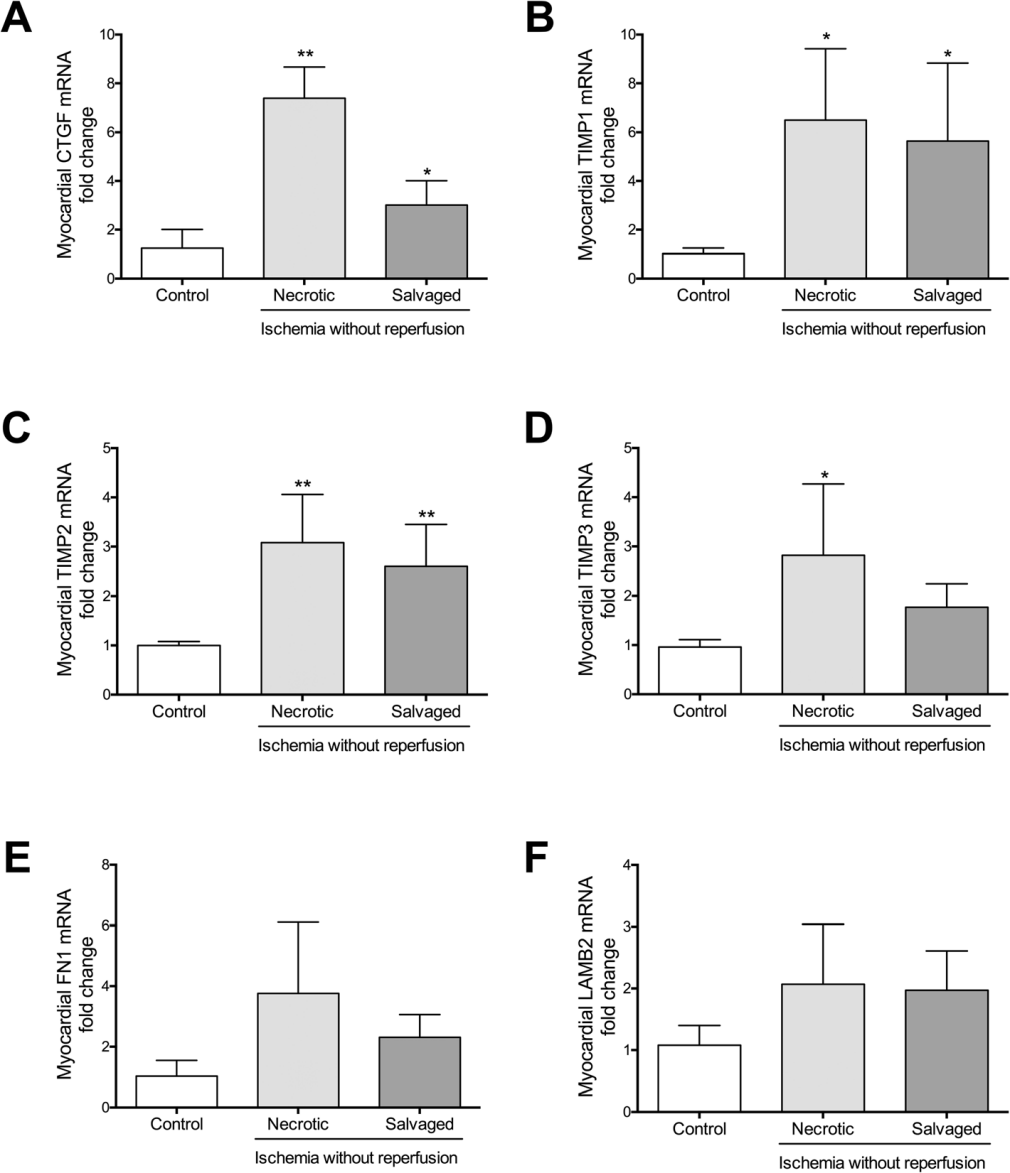
mRNA expression of key genes implicated in matrix remodeling in the necrotic and salvaged myocardium isolated after 90-min of ischemia without reperfusion. The gene expression of connective tissue growth factor (CTGF, **a**), tissue inhibitors of metalloproteinases (TIMP)1 (**b**), TIMP2 (**c**), and TIMP3 (**d**) as well as fibronectin (FN, E) and laminin (LAMB, F) was determined in the necrotic and salvaged myocardium from the 90-min of ischemia without reperfusion group and was compared to control group. Data (mean ± SD, *n* ≥ 5) were analysed by one-way ANOVA analysis followed by Bonferroni test. *P < 0.05, **P < 0.01 vs. control group

Analyzing the ground substance in both necrotic and salvaged areas, we detected higher levels of laminin and fibronectin (Fig. 1c and d, respectively), mainly present in the basement membrane of the cardiomyocytes (laminin) or surrounding the fascicles of the cardiac muscular cells (fibronectin). Moreover, a tendency to increased laminin (Fig. 2e) and fibronectin (Fig. 2f) mRNA expression was detected in necrotic and salvaged areas isolated from the ischemia group in comparison to healthy hearts (Table 3).

According to these results, ECM remodeling begins soon after ischemia onset and is similar irrespective of whether myocardial tissue becomes necrotic or salvaged.

### Dynamics of key genes involved in ECM remodeling in the necrotic and salvaged myocardium after 90-min of ischemia without reperfusion

The mRNA levels of tissue inhibitors of metalloproteinases (TIMP)1, and TIMP2 (Fig. 2b and Fig. 2c, respectively), but not TIMP4, were upregulated in both regions (necrotic and salvaged) after 90 min of ischemia (without reperfusion) (Table 3). TIMP3 mRNA expression was only elevated in necrotic but not salvaged myocardium after severe ischemia (Fig. 2d). However, expression of metalloproteinases (MMP)2, MMP9, MMP15, and MMP16 was not altered in either necrotic or salvaged regions compared to control myocardium, whereas MMP14 were significantly augmented in the salvaged, but not in the necrotic, myocardium (Table 3). Ultimately, when analyzing key transcription factors involved in ECM remodeling, mRNA levels of connective tissue growth factor (CTGF) (Fig. 2a), but not tumor necrosis factor (TNF)-α, basigin (BSG), or transforming growth factor (TGF)-β, were heightened in the necrotic and salvaged areas isolated from no reperfusion group (Table 3).

As with the ECM components, expression of gene factors participating in ECM remodeling was comparable in both regions after 90-min of ischemia. Therefore, prior to coronary reperfusion, the interstitium of the whole area at risk (comprising the future necrotic and salvaged myocardium) concurrently changes during ischemia.

### ECM dynamics in the necrotic and salvaged myocardium isolated one week or one month after coronary reperfusion

At macroscopic level, 1 week after coronary revascularization, no differences in left ventricular wall thickness was detected between necrotic and salvaged regions. However, at chronic (1-month) phase, a significant reduction in myocardial wall thickness was observed in the necrotic area compared to salvaged area (Table 1).

#### Fibrilar component of ECM

**Fig. 3:**
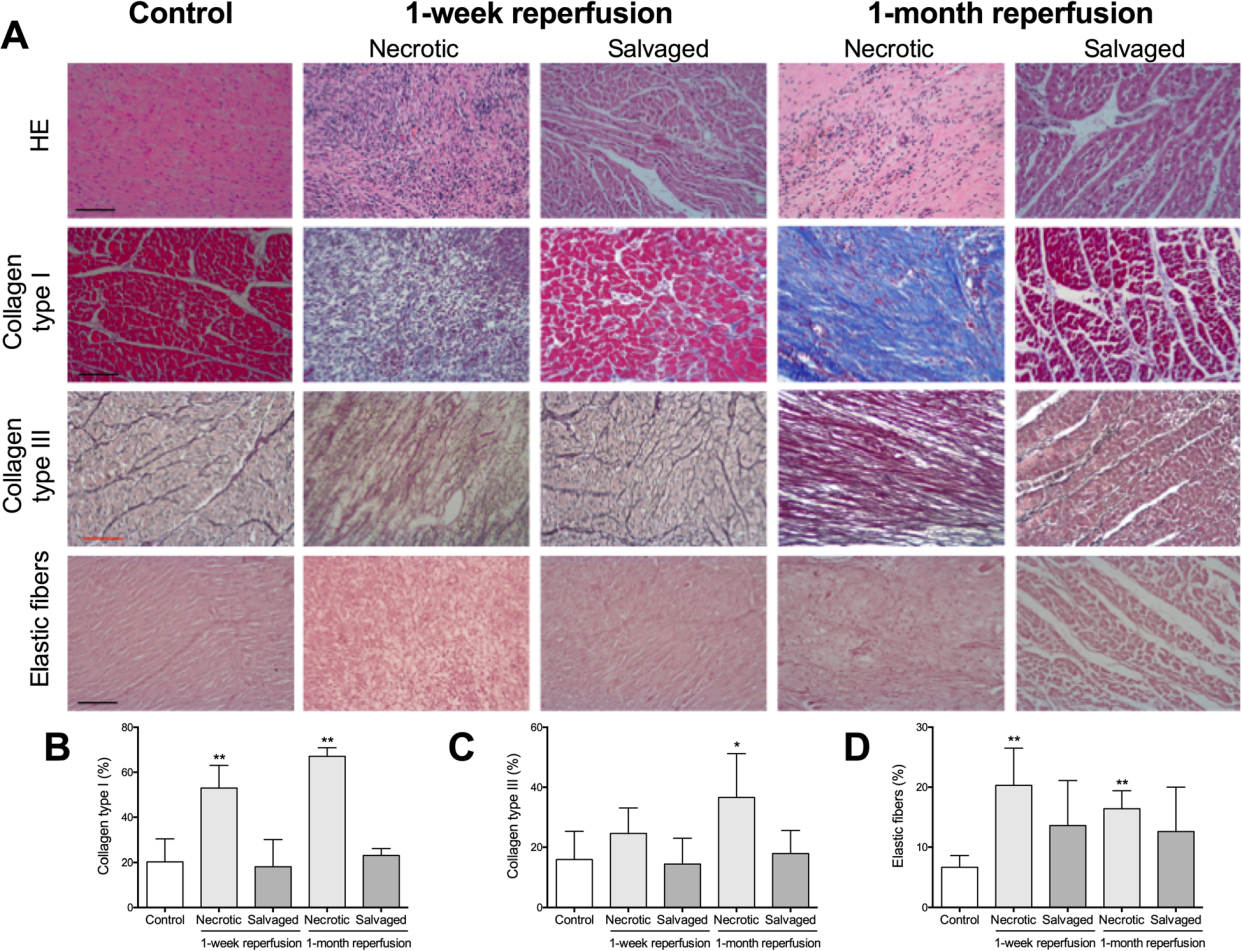
Morphometric analysis of extracellular matrix fibers from the necrotic and salvaged myocardium isolated at one week and one month after coronary reperfusion. (**a**) Representative images from control group (left panel) and from necrotic and salvaged myocardium isolated from the two reperfused myocardial infarction (MI) groups [90-min ischemia followed by 1-week (middle panel) and 1-month (right panel) reperfusion] stained with hematoxylin-eosin (HE) (upper panel), Masson’s trichrome (upper-middle panel), Gomori’s reticulin (lower-middle panel) and orcein for elastic fibers (lower panel). The scale bars indicate 50 μm. Morphometric quantification of collagen type I (**b**), collagen type III (**c**) and elastic fibers (**d**). Images were analyzed with Image-Pro Plus analysis software in a blinded fashion. The area occupied by collagen type I, collagen type III and elastic fibers was greater in the necrotic, but not in the salvaged, myocardium from both reperfused MI model in comparison to control tissue. Data (mean ± SD, *n* ≥ 5) were analysed by one-way ANOVA followed by Bonferroni test. Scoring was performed by a blinded observer unaware of the experimental group. *P < 0.05, **P < 0.01 vs. control

In the 1-week reperfusion group, a larger amount of collagen type I (Fig. 3b) and elastic fibers (Fig. 3d) was detected in the necrotic region, while a tendency to increase was observed in collagen type III (Fig. 3c). In the necrotic myocardium from the 1-month reperfusion group, the collagen type I (Fig. 3b), collagen type III (Fig. 3c), and elastic fibers (Fig. 3d) content was significantly elevated in comparison to control hearts (Table 2). In contrast, salvaged myocardium from both reperfused MI groups was barely increased in these components and their values were similar to a healthy myocardium (Fig. 3, Table 3).

Subsequently, the organization of both types of collagen fibers (Fig. S2A) in the two areas was evaluated using Fast Fourier transform. The diagrams obtained from the necrotic area were elongated, while the rounded diagrams were found in images from salvaged regions of both (1-week and 1-month) reperfusion groups and myocardium isolated from the control group. This indicates that collagen types I and III (Fig. S2B and Fig. S2C, respectively) displayed a more organized pattern in the necrotic tissue than in the salvaged myocardium, the latter organization being comparable to a control heart.

**Fig. 4:**
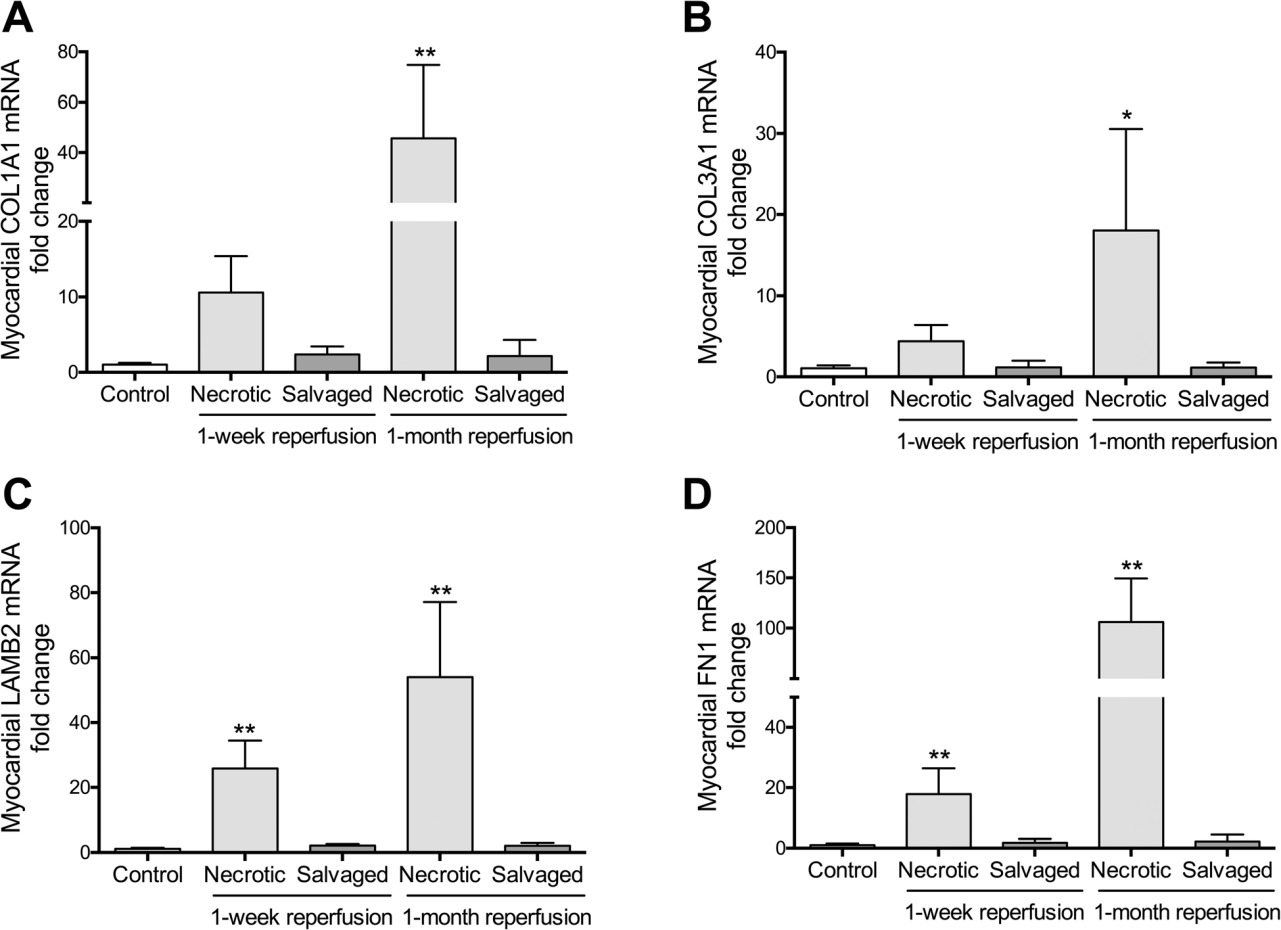
mRNA expression of key proteins participating in matrix composition in the necrotic and salvaged myocardium isolated one week and one month after coronary reperfusion. The gene expression of collagen (COL) type I (**a**) and type III (**b**) as well as fibronectin (FN, **c**) and laminin (LAMB, **d**) was assessed in the necrotic and salvaged myocardium from the 1-week and 1-month reperfusion groups and was compared to control group. Data (mean ± SD, *n* ≥ 5) were analysed by one-way ANOVA analysis followed by Bonferroni test. *P < 0.05, **P < 0.01 vs. control group

Moreover, compared to the weak gene expression in controls, mRNA expression of the two collagen types increased in the necrotic, but not in the salvaged, myocardium of both reperfused MI groups (Fig. 4a, Fig. 4b and Table 3). Taken together, these results suggest that not only the quantity, but also the organization and mRNA expression of both collagen type fibers differ between necrotic and salvaged myocardium, whose pattern was similar to control tissue.

#### Ground substance

**Fig. 5:**
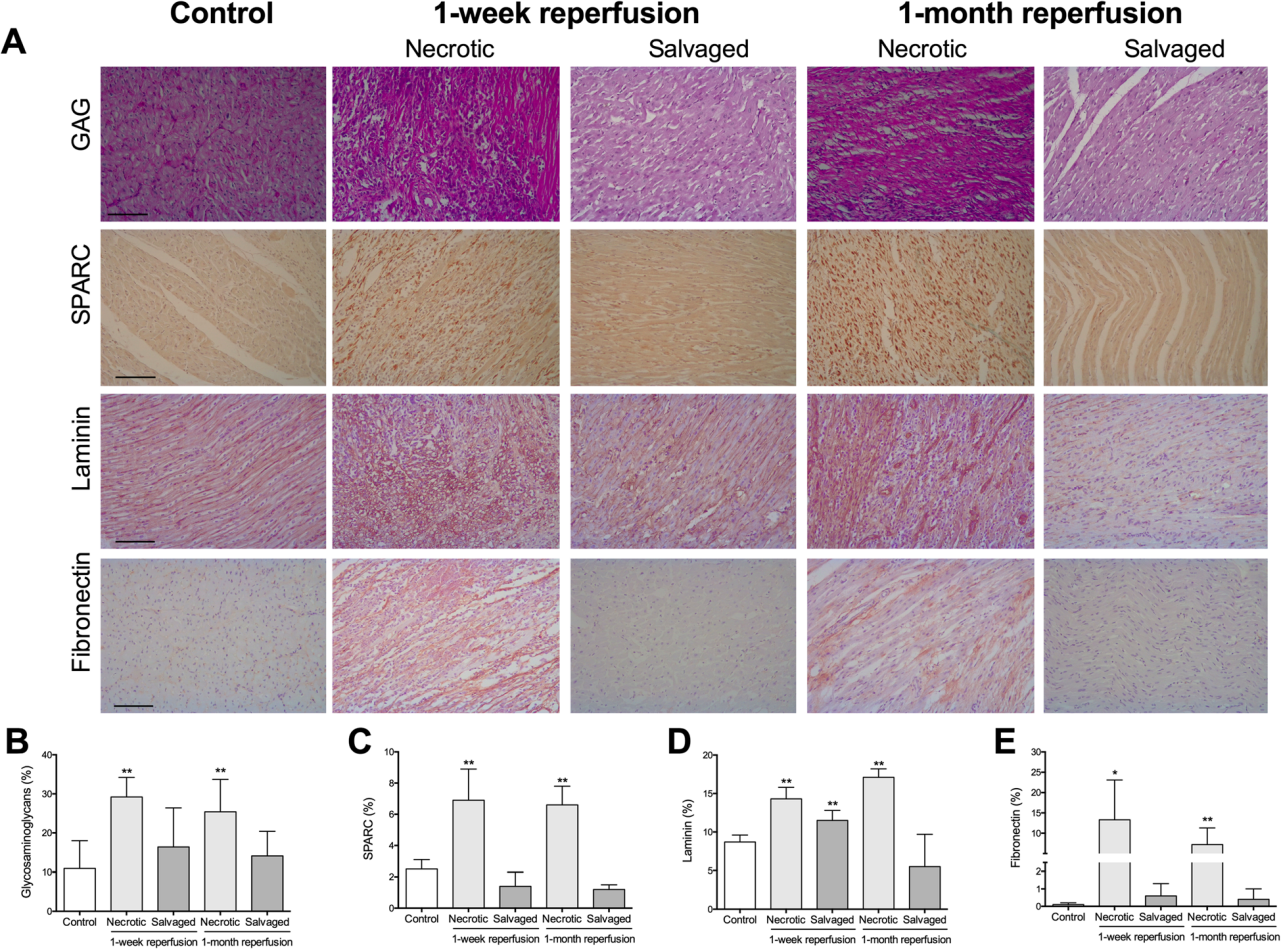
Morphometric analysis of ground substance elements from the necrotic and salvaged myocardium isolated one week and one month after coronary reperfusion. (**a**) Representative images from control group (left panel) and from the necrotic and salvage myocardium isolated from the two reperfused myocardial infarction (MI) groups [90-min ischemia, then one week (middle panel) and one month (right panel) reperfusion], stained with periodic acid–Schiff for glycosaminoglycans (GAG; upper panel) as well as with the specific marker for secreted protein acidic and rich in cysteine (SPARC, upper-middle panel), laminin (lower-middle panel) and fibronectin (lower panel). The scale bars indicate 50 μm. Morphometric quantification of GAG (**b**), SPARC (**c**), laminin (**d**), and fibronectin (**e**). Images were analyzed with Image-Pro Plus analysis software. The area occupied by GAG, SPARC, laminin and fibronectin was increased in the necrotic, but not in the salvaged, myocardium from reperfused MI groups in comparison to the control myocardium. Data (mean ± SD, *n* ≥ 5) were analysed by one-way ANOVA followed by Bonferroni test. Scoring was performed by a blinded observer unaware of the experimental group. *P < 0.05, **P < 0.01 vs. control

An augmented content of GAG (Fig. 5a), mainly located in the space left by the necrotic cardiomyocytes, was detected in the necrotic, but not in the salvaged, myocardium isolated from the 1-week and 1-month reperfusion groups (Fig. 5b, Table 2).

Analyzing adhesion glycoproteins, increased SPARC (Fig. 5c), laminin (Fig. 5d), and fibronectin (Fig. 5e) content was observed in the necrotic but not salvaged myocardium isolated from both reperfused MI models. The augmented laminin content was detected mainly surrounding the necrotic cardiomyocytes (1-week after reperfusion) or the basement membrane of the blood vessels (1-month after reperfusion), while fibronectin and SPARC were located in the interstitial space left by necrotic cardiomyocytes from the necrotic area.

Furthermore, mRNA expression of laminin (Fig. 4c) and fibronectin (Fig. 4d) was elevated in the necrotic myocardial samples from the 1-week and 1-month reperfusion groups compared to the corresponding salvaged tissue, whose levels were similar to controls (Table 3).

In summary, these results point to necrotic and salvaged myocardium as having a completely different interstitium after coronary reperfusion, with the salvaged myocardium composition comparable to control hearts.

### Dynamics of genes involved in ECM remodeling in necrotic and salvaged areas after coronary reperfusion

**Fig. 6:**
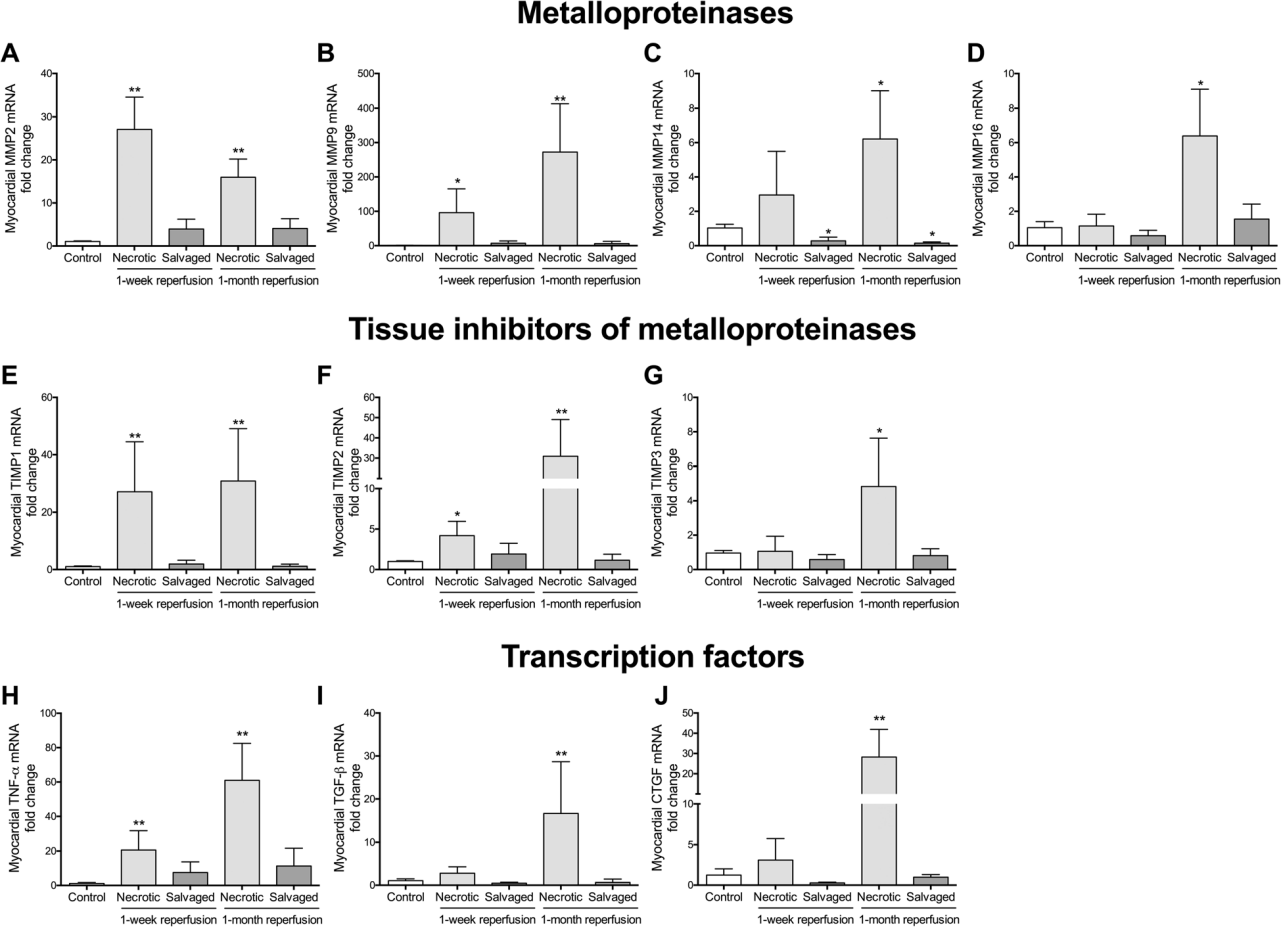
mRNA expression of central factors implicated in regulating extracellular matrix remodeling in the necrotic and salvaged myocardium isolated one week and one month after coronary reperfusion. The gene expression of metalloproteinase (MMP)2 (**a**), MMP9 (**b**), MMP14 (**c**), MMP16 (**d**), tissue inhibitors of metalloproteinases (TIMP)1 (**e**), TIMP2 (**f**), TIMP3 (**g**) as well as the transcription factors tumor necrosis factor (TNF)-α (**h**), transforming growth factor (TGF)-β (**i**), and connective tissue growth factor (CTGF, **j**) was assessed in the necrotic and salvaged myocardium from the 1-week and 1-month reperfusion groups and was compared to control group. Data (mean ± SD, *n* ≥ 5) were analysed by one-way ANOVA analysis followed by Bonferroni test. *P < 0.05, **P < 0.01 vs. control group

mRNA levels of TIMP1 and TIMP2 (Fig. 6e and Fig. 6f, respectively), but not TIMP4, remained higher than controls in the necrotic area of both reperfused MI groups (Table 3). TIMP3 gene expression was only heightened in the 1-month reperfusion group (Fig. 6g). However, even though mRNA expression was increased after 90 min of ischemia in the salvaged myocardium, coronary reperfusion reduced the four evaluated TIMPs to baseline levels or even lower (Table 3).

Although mRNA expression of MMP2 (Fig. 6a) and MMP9 (Fig. 6b) was substantially increased in the necrotic tissue from both reperfused MI groups, MMP14 (Fig. 6c) and MMP16 (Fig. 6d) levels were only significantly augmented in the 1-month reperfusion group. Conversely, in the salvaged area mRNA expression were normal or even lower than control tissue (Table 3).

Ultimately, in the necrotic myocardium, upregulation of TNF-α (Fig. 6h) and deregulation of BSG were observed 1 week after MI induction, whereas levels of TNF-α, CTGF (Fig. 6i), and TGF-β (Fig. 6j) were elevated after 1 month. Analyzing the salvaged area from both reperfused MI groups, BSG deregulation was detected in the 1-week reperfusion group, but mRNA levels of the remaining evaluated transcription factors were unaltered (Table 3).

Parallel to the morphometric results, expression of key genes involved in ECM remodeling was different in necrotic and salvaged myocardium after coronary reperfusion. Consequently, despite interstitial changes occurred concomitantly in necrotic and salvaged myocardium during ischemia, the resulting ECM composition after myocardial repair differed between necrotic and salvaged myocardium.

## Discussion

After morphometric and molecular analysis, we concluded that necrotic and salvaged myocardium display similar ECM remodeling during ischemia. However, after coronary reperfusion necrotic myocardium develops into fibrotic scar, while different regulatory mechanisms cause matrix from salvaged areas to be restored to healthy tissue. Therefore, coronary reperfusion exerts beneficial effects not only in reducing cardiomyocyte apoptosis but also in maintaining the proper ECM composition.

### Role of myocardial matrix in pathophysiology after MI

ECM is a non-cellular, three-dimensional network made up of fibers and ground substance, mainly comprising GAG, proteoglycans and adhesion glycoproteins. Matrix components regulate diverse cellular processes such as survival, migration, proliferation, apoptosis, and inflammatory responses [2, 3, 10] and undergo remodeling in both physiological and pathological conditions.

MI is caused by the thrombotic occlusion of a coronary artery. Current gold-standard therapy for ST-segment elevation MI patients is coronary reperfusion to reestablish nutrient and oxygen supply in the downstream myocardium [4]. Complete and rapid revascularization of the culprit artery, ideally by percutaneous coronary intervention, increases salvaged myocardium, consequently enhancing patient prognosis [5–7].

Even though percutaneous intervention has revolutionised management and outcome in MI patients, heart failure is indisputably a pivotal player in late morbi-mortality and post-MI patient healthcare costs. Incidence of heart failure after MI ranges from 14 to 36% and is a multifactorial phenomenon leading to adverse left ventricular geometry and wall thinning [8, 9]. Since matrix remodelling is essential to preserve cardiac structure, adequate timely and localized myocardial ECM remodelling is mandatory [3, 10–12]. The importance of prompt coronary revascularization in minimizing cardiomyocyte damage has been widely addressed [5], but little attention has been paid to its role in preserving myocardial matrix, which is potentially crucial to prevent adverse ventricular remodeling and heart failure development.

Given all this, we aimed to gain insight into myocardial ECM variations throughout the ischemia and reperfusion process following MI using morphometric and genetic analysis to pinpoint the mechanisms underlying the progression of post-MI adverse ventricular remodeling.

### ECM remodeling begins soon after ischemia onset in both necrotic and salvaged myocardium to form a provisional matrix

Rapid modifications in ECM composition need to occur for the infarcted heart to cope with mechanical and metabolic stress due to the ischemic environment. Indeed, impairment of cardiomyocyte physiology and apoptosis activation are reported to begin soon after ischemia onset [18]. Therefore, forming a provisional matrix prior to the definitive fibrotic scar is crucial to ensure adequate cardiac structure and functionality during hypoxia and also to provide a scaffold for reparative cells such as migrating leukocytes, proliferating fibroblasts, and endothelial cells [2, 3].

According to our results, there were more laminin and fibronectin adhesion glycoproteins both in necrotic and salvaged myocardium in the infarct area after 90 min of ischemia (without reperfusion). Increased vascular permeability and edema presence in the infarcted tissue have been extensively described soon after MI induction, hence plasma-derived proteins are proposed to form this provisional interstitium [2]. In fact, acute MI patients displayed increased circulating levels of laminin and fibronectin [1, 19], while myocardial mRNA levels were similar to control tissue, indicating that ischemic hearts were not able to secrete these two proteins. Consequently, the increased circulating levels of both glycoproteins together with the presence of myocardial edema could explain the laminin and fibronectin deposition in the myocardial interstitium during the hypoxic phase.

Laminin is an essential component of the cardiomyocyte basement membrane responsible for anchoring cells to the ECM and binding receptor proteins, whereas fibronectin maintains the tensile strength of the necrotic tissue, and regulates cellular adhesion and migration [2, 3, 20]. In the necrotic and salvaged area from the no reperfusion group, laminin and fibronectin are located surrounding cardiomyocyte cells (endomisium) or muscular fascicles (perimisium), respectively. An elevated presence of fibronectin surrounding necrotic cardiomyocytes few hours after ischemia onset has also been confirmed in a rodent non-reperfused MI model [21]. Since both glycoproteins are responsible for cellular adhesion, their rapid presence immediately after ischemia onset might reflect a defense mechanism in the whole jeopardized area of the infarcted heart to preserve the myocardial architecture due to the beginning of cardiomyocyte necrosis.

Molecular analysis revealed an increase in TIMP1, TIMP2, and TIMP3 mRNA expression soon after ischemia onset in the whole area at risk, while no variations in the evaluated MMPs were observed. In line with our hypothesis, ECM degradation could be initially blocked due to rapid upregulation of TIMP with the objective of impeding the effects of endogeneous MMP and maintaining the myocardial structure in the hyper-acute phase post-MI. In the MI scenario, TIMPs are suggested to help avoid excessive ECM accumulation, which leads ultimately to adverse systolic dysfunction [20, 22]. However, to our knowledge, this is the first study demonstrating early upregulation of TIMPs soon after ischemia onset, probably as a protective mechanism to block early degradation of myocardial ECM.

In this context, CTGF, matricellular protein secreted by damage cardiomyocytes, could be the major driver due to its heightened gene expression in both necrotic and salvaged myocardium from the no reperfusion group. Although this factor has traditionally participated in fibrosis development in late phases post-MI, its role soon after MI induction was also demonstrated since transgenic mice overexpressing CTGF and submitted to permanent coronary artery ligation displayed reduced infarct area even 1 day after MI induction [23].

In summary, morphometric and genetic changes in the myocardial interstitium occur soon after ischemia onset in the whole area at risk (necrotic and salvaged myocardium). This provisional matrix could play a role in protecting the myocardium from the hypoxic environment, ensuring correct cardiac function despite apoptotic cardiomyocytes, and facilitating cell regeneration.

### Interstitium from salvaged myocardium initially changes during ischemia but is restored to control myocardial ECM composition after coronary reperfusion

Salvaged myocardium is defined as the difference between the area at risk during acute coronary occlusion and the final area of necrosis [24]. The greater the area of salvaged myocardium, the more conserved the systolic function and the better the reported prognosis [5, 25]. Since saving cardiomyocytes together with escaping from depressed systolic function due to a proper matrix composition is essential to maintain left ventricular structure, we sought to gain insight into fluctuations in the ECM composition in the salvaged myocardium.

According to our results, in the first minutes of ischemia (prior to coronary reperfusion), ECM composition behaves in a similar way in both the necrotic and salvaged myocardial areas. However, following revascularization, even though the infarct area develops to form the definitive fibrotic scar mainly comprised by collagen deposition, the ECM composition from the salvaged myocardium is comparable to a control heart.

These dynamics were observed not only in morphometric but also in the genetic analysis of the pivotal factors involved in ECM remodeling. After ischemia, upregulated expression of tissue inhibitors TIMP1, TIMP2, TIMP3 and CTGF was observed in both the necrotic and salvaged myocardium. However, following myocardial reperfusion, mRNA expression of TIMP1, TIMP2, and TIMP3 as well as metalloproteinase-2 and metalloproteinase-9 was enhanced only in the necrotic but not in the salvaged myocardium.

Rapid coronary revascularization has been indisputably linked with a greater area of viable myocardium, thus reducing cardiomyocyte apoptosis, infarct size and adverse cardiovascular events [5–7]. However, in our study, revascularization is shown to exert beneficial effects not only in myocardial salvage but also in maintaining an adequate myocardial matrix composition.

Salvaged myocardium seems to suffer reversible ECM remodeling induced by transient nonlethal ischemia, but eventually reverts to normal status as long as coronary blood flow is restored. As an auto-regulatory mechanism to protect the heart from ischemia, the whole myocardium at risk undergoes the same ECM changes, irrespective of whether it progress further to necrotic or salvaged myocardium. Therefore, currently recommended early epicardial coronary reperfusion can exert a beneficial effect on well-established myocardial salvage but also in preserving matrix composition, thus reducing infarct size and enhancing patient outcomes.

## Conclusions

ECM changes start soon after ischemia onset, as reflected by early higher levels of fibronectin, laminin, and elastic fibers. Despite matrix remodeling concomitantly occurs in both necrotic and salvaged myocardium after prolonged ischemia, matrix composition in the salvaged area after coronary reperfusion resembles the control heart. Therefore, rapid coronary reperfusion is essential not only to save cardiomyocytes but also to preserve matrix, thus avoiding impaired left ventricular remodeling.

## Methods

### Experimental protocol

This study adheres to the ARRIVE guidelines and was approved by the Institutional Animal Care and Use Committee of the University of Valencia and conforms with the Guide for the Care and Use of Laboratory Animals published by the US National Institutes of Health (NIH Publication No. 85–23, revised 1993), as well as European (2010/63/EC) and national regulations (RD53/2013). Juvenile domestic female pigs weighing 25–30 kg were included in the experimental study. Animals were obtained from the local farm “El Pampo” (Registration number: ES462440000003).

After engagement of the proximal left anterior descending (LAD) artery using a trans-femoral 6F Amplatz Left 0.75 catheter, an angioplasty balloon (2.5 × 16 mm) was placed in the mid-LAD and used to induce severe coronary ischemia by transitory (90-min) balloon inflation.

The complete experimental protocol has been previously validated and can be consulted elsewhere [13, 14].

### Experimental groups

One control group and three independent MI experimental groups were formed. In the MI groups, after 90-min occlusion of the mid-LAD by the angioplasty balloon, experiments were categorized as: 1) without reperfusion or followed by 2) 1-week or 3) 1-month reperfusion (*n* = 5 each). The control group (*n* = 5) was subjected to the same experimental protocol used in the MI groups, but the angioplasty balloon was not inflated and thus ischemia and infarction were not induced.

### Macroscopic analysis of myocardial samples

In order to delimit the area at risk (defined as the myocardium that is jeopardized by the coronary occlusion), 20 ml of 4% thioflavin-S solution (Sigma Aldrich, MO) was infused through the lumen of the inflated over-the-wire balloon immediately before sacrifice. In the no reperfusion group thioflavin-S was administered at the end of the 90-min ischemia period. In the 1-week and 1-month reperfusion groups, prior to sacrifice of the animal, the angioplasty balloon was re-inflated at the same location used to induce the infarction (mid-LAD) and the colorant was injected through the lumen of the inflated balloon. Subsequently, anesthetized animals were euthanized by intravenous administration of potassium chloride (0.9%, 2 ml) and then heart was excised [13, 14].

Once the heart was excised, the left ventricle was sectioned into 5-mm thick short-axis slices. Firstly, each slide was viewed under ultraviolet light and photographed. Light blue represents thioflavin-S myocardial uptake after infusion into the area at risk, while dark blue indicates lack of perfusion (Fig. S3).

Secondly, to determine the extent of the infarcted area, slices were incubated in 2% 2,3,5-triphenyltetrazolium chloride (Sigma Aldrich, MO) solution at 37 °C for 20 min. Afterwards they were viewed under room light and photographed.

Necrotic and salvaged areas within the left ventricle were defined in all short-axis slices. Necrotic area was regarded as myocardium located in the area at risk that did not stain with triphenyltetrazolium (thioflavin-S+ and triphenyltetrazolium-). Salvaged myocardium was defined as the myocardium located in the area at risk that stained with triphenyltetrazolium (thioflavin-S+ and triphenyltetrazolium+) (Fig. S3).

### Microscopic analysis and immunohistochemistry of myocardial samples

All samples were fixed in 4% paraformaldehide, embedded in paraffin, sectioned and mounted on double gelatin-coated glass slides. Hematoxylin and eosin stain was utilized for histological analysis. To further characterize myocardial samples, orcein stain for elastic fibers, Masson’s trichrome stain for collagen type I, reticulin for collagen type III, and periodic acid–Schiff for glycosaminoglycan were also performed.

### Immunohistochemistry

For immunohistochemistry, after peroxidase inactivation (H2O2 0.3%) and blockade with horse serum, sections were incubated overnight (4 °C) with the specific primary antibodies diluted in PBS/BSA 0.1%. Specific labelling was detected with a biotin-conjugated goat anti-mouse or goat anti-rabbit secondary antibody (1:500 dilution, Dako Glostrup, Denmark) [14]. Further information about the primary antibodies, antigen retrieval method, and antibody concentration is detailed in Table 4.

**Table 4:** Summary of the primary antibodies data (concentration, antigen retrieval method, reference) employed in this study

Marker	Concentration	Antigen retrieval	Reference
Laminin	1:50	Enzymatic	Abcam (ab11575)
Fibronectin	1:150	Enzymatic	Abcam (#ab23751)
SPARC	1:100	pH low	Antibodies-online (#ABIN1092072)
Perlecan	2 μg/ml	pH low	LSBio (#LS-C390348)

Abbreviation: *SPARC* Secreted protein acidic and rich in cysteine

### Morphometric quantification of extracellular matrix components in the infarcted myocardium

For each sample and each stain, eight photographs at 20x magnification were taken in independent fields using an optical microscope Leica DM3000 (Leica Microsystems, Wetzlar, Germany). Images were morphometrically analysed using Image ProPlus 7.0 software (Media Cybernetics Inc., Rockville, MD) performed in a blinded manner on coded slides [13, 14].

### Fourier transformation of cardiac images

Previous information about the protocol for quantifying the organization of collagen fibers can be consulted elsewhere [13] and has been previously validated by [26]. Briefly, collagen fibers orientation index is the ratio of the maximum width (minor axis) and the maximum length (major axis) of the threshold Fourier 2D power plot. A completely random orientation, suggesting total disorganization, results in an organization index that approximates to 1, whereas a perfect organization results in an organization index close to 0. Therefore, higher values for the orientation index indicate disorganization and lower values a more parallel organization [13, 26].

### Quantitative real-time polymerase chain reaction

In order to extract RNA, RNeasy Plus Mini Kit (QIAGEN GmbH, Hilden, Germany) was employed following the manufacturer’s instructions. Gene expression was determined by real time Polymerase Chain Reaction using a 7900HT Fast Real-Time Polymerase Chain Reaction System (Applied Biosystems, Thermo Fisher Scientific, Waltham, MA). The values of the threshold cycle (Ct) were calculated by triplicate and normalized to the housekeeping gene GAPDH [14].

We used specific primers pre-designed by Bio-Rad Laboratories (Hercules, CA) for analysis of porcine samples. Further information about the primers employed in this study is specified in Table 5.

**Table 5:** References of the primers utilized in this study

Gene	Reference	Gene	Reference
TNF-α	qSscCID0014087	TIMP1	qSscCED0019748
BSG	qSscCED0014523	TIMP2	qSscCED0008300
TGF-β	qSscCID0018090	TIMP3	qSscCED0010478
CTGF	qSscCED0011014	TIMP4	qSscCID0004859
MMP2	qSscCID0011742	COL1A1	qSscCED0020342
MMP9	qSscCID003617	COL3A1	qSscCID0003467
MMP14	qSscCED0016833	FN1	qSscCID0003939
MMP15	qSscCID0012408	LAMB2	qSscCED0011174
MMP16	qSscCID0002889	GAPDH	qSscCED0017494

Abbreviations: *BSG* Basigin; *COL* Collagen; *CTGF* Connective tissue growth factor; *FN* Fibronectin; *LAMB* Laminin; *MMP* Metalloproteinases; *TGF* Transforming growth factor; *TIMP* Tissue inhibitors of metalloproteinases: *TNF* Tumor necrosis factor

### Statistical analysis

Continuous variables were expressed as the mean ± SD. One-way ANOVA analysis or unpaired t-Students’ test was used for comparisons and statistical significance was considered for two-tailed *p*-value less than 0.05. SPSS 19.0 (SPSS, Inc., Chicago, IL) was used throughout. All measurements were performed by researchers unaware of the experimental group.

## Supplementary information

**Additional file 1: Supplementary Figure 1.** Type I and type III collagen fiber organization in the necrotic and salvaged myocardium isolated after 90-min of ischemia. (A) Representative images from control group (left panel) and the necrotic (central panel) and salvaged (right panel) myocardium isolated from the severe ischemia group (90-min ischemia without reperfusion, right panel) stained with Masson’s trichrome (upper panels) and Gomori’s reticulin (lower panels) and the Fourier transform spectra obtained from these images. Images were analyzed with Image-Pro Plus analysis software. The scale bars indicate 50 μm. No differences existed between the collagen organization index of the salvaged and necrotic tissue from the severe ischemia group and control when compared with the organization of collagen type I (B) and type III (C). Upper and lower lines of the boxes represent the 25th and 75th percentiles. Data were analysed by non-paired t-Student’s test. Scoring was performed by a blinded observer unaware of the experimental group.

**Additional file 2: Supplementary Figure 2.** Type I and type III collagen fiber organization in the necrotic and salvaged myocardium isolated at one week and one month after coronary reperfusion. (A) Representative images from control group (left panel) and from the necrotic and salvage myocardium isolated from the two reperfused myocardial infarction (MI) groups [90-min ischemia followed by 1-week (middle panel) or 1-month (right panel) reperfusion] stained with Masson’s trichrome (upper panel) and Gomori’s reticulin (lower panel) and the Fourier transform spectra obtained from these images. Images were analyzed with Image-Pro Plus analysis software. The scale bars indicate 50 μm. The collagen type I (B) and type III (C) organization index was lower in the necrotic, but not in the salvaged, myocardium from the one-week and one-month reperfusion groups in comparison to the control myocardium. Data were analysed by non-paired t-Student’s test. Scoring was performed by a blinded observer unaware of the experimental group. *P < 0.05, **P < 0.01 vs. control.

**Additional file 3: Supplementary Figure 3.** Macroscopic study of myocardial hearts obtained from the swine model. Samples were stained with thioflavin-S (T-S, left) and 2,3,5-triphenyltetrazolium chloride (TTZ, right). Necrotic tissue was defined as the myocardial area stained with TTZ. Salvaged myocardium was defined as the non-infarcted territory within the area at risk clearly outside the infarcted area (with TTZ and T-S staining).

## Data Availability

The datasets used and/or analysed during the current study are available from the corresponding author on reasonable request.

## Abbreviations

BSG: Basigin
CTGF: Connective tissue growth factor
ECM: Extracellular matrix
GAG: Glycosaminoglycans
LAD: Left anterior descending
MI: Myocardial infarction
MMP: Metalloproteinases
TIMP: Tissue inhibitors of metalloproteinases
TGF-β: Transforming growth factor-β
TNF-α: Tumor necrosis factor-α
