# Supplementary figures and images for: Interstitial changes after reperfused myocardial infarction in swine: morphometric and genetic analysis
Source: BMC Vet Res. 2020 Jul 29;16:262. doi: 10.1186/s12917-020-02465-6 (PMC7388500; doi:10.1186/s12917-020-02465-6)

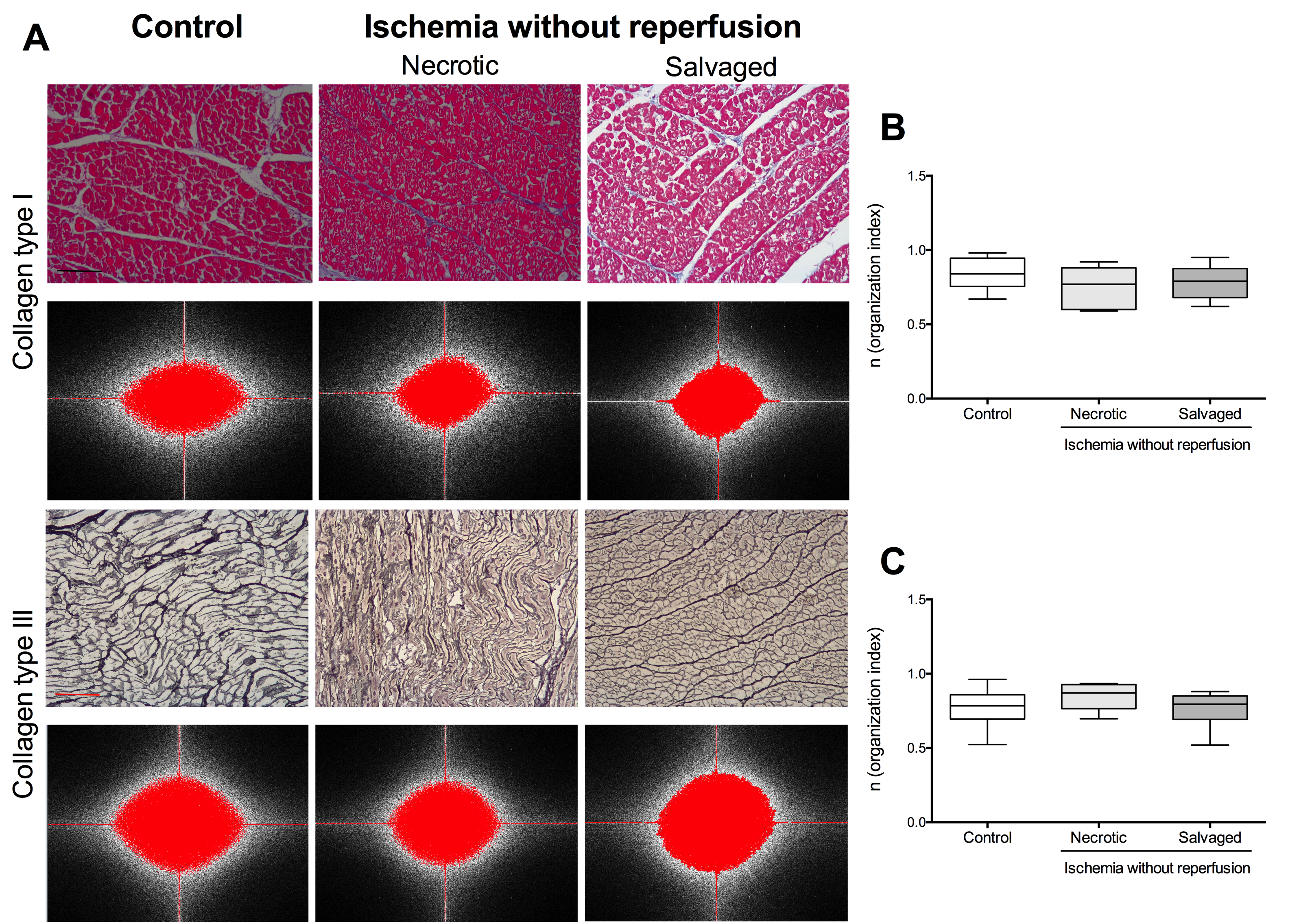

Supplement: Supplementary file 1 — Additional file 1: Supplementary Figure 1. Type I and type III collagen fiber organization in the necrotic and salvaged myocardium isolated after 90-min of ischemia. (A) Representative images from control group (left panel) and the necrotic (central panel) and salvaged (right panel) myocardium isolated from the severe ischemia group (90-min ischemia without reperfusion, right panel) stained with Masson’s trichrome (upper panels) and Gomori’s reticulin (lower panels) and the Fourier transform spectra obtained from these images. Images were analyzed with Image-Pro Plus analysis software. The scale bars indicate 50 μm. No differences existed between the collagen organization index of the salvaged and necrotic tissue from the severe ischemia group and control when compared with the organization of collagen type I (B) and type III (C). Upper and lower lines of the boxes represent the 25th and 75th percentiles. Data were analysed by non-paired t-Student’s test. Scoring was performed by a blinded observer unaware of the experimental group. [file 12917_2020_2465_MOESM1_ESM.tiff]

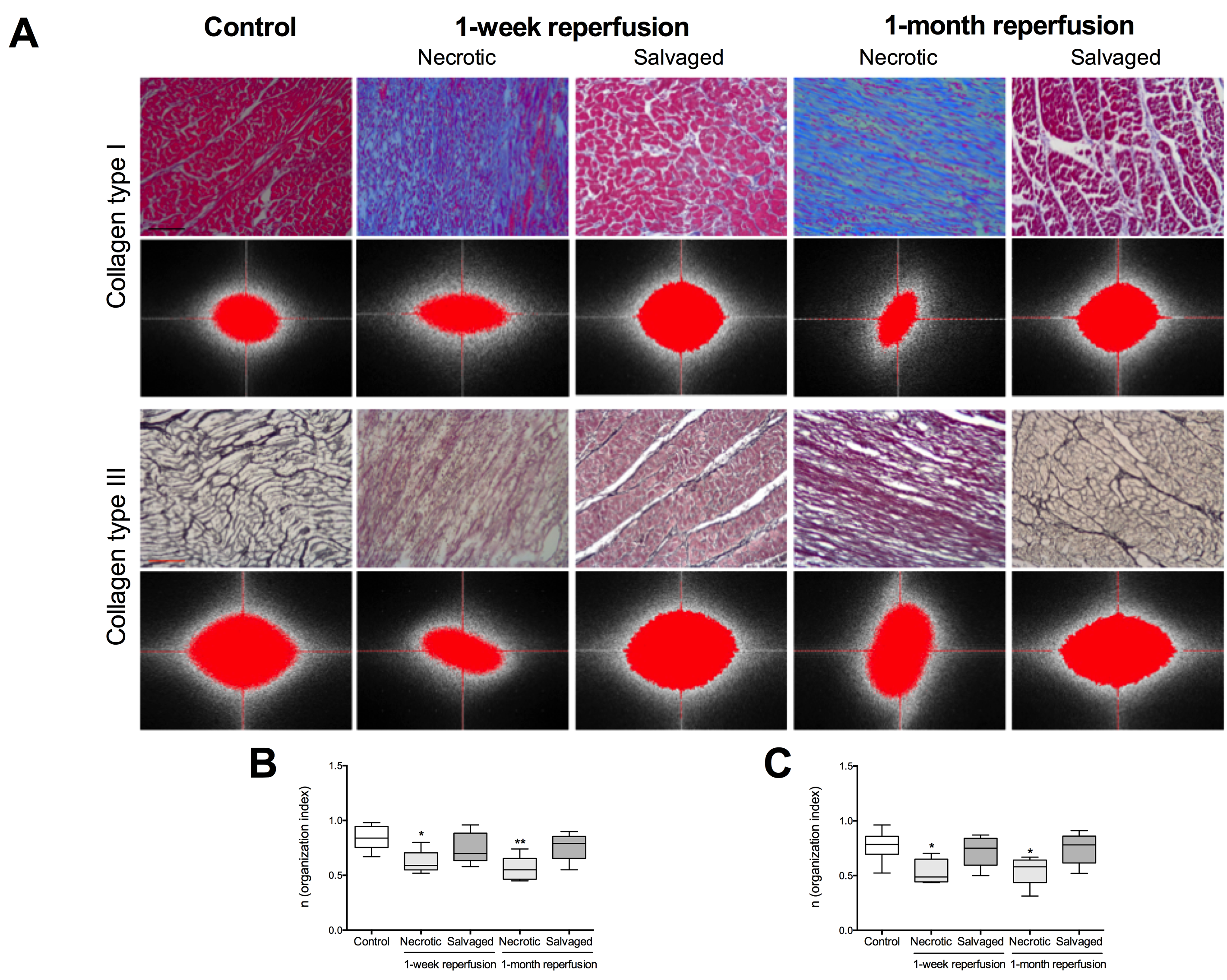

Supplement: Supplementary file 2 — Additional file 2: Supplementary Figure 2. Type I and type III collagen fiber organization in the necrotic and salvaged myocardium isolated at one week and one month after coronary reperfusion. (A) Representative images from control group (left panel) and from the necrotic and salvage myocardium isolated from the two reperfused myocardial infarction (MI) groups [90-min ischemia followed by 1-week (middle panel) or 1-month (right panel) reperfusion] stained with Masson’s trichrome (upper panel) and Gomori’s reticulin (lower panel) and the Fourier transform spectra obtained from these images. Images were analyzed with Image-Pro Plus analysis software. The scale bars indicate 50 μm. The collagen type I (B) and type III (C) organization index was lower in the necrotic, but not in the salvaged, myocardium from the one-week and one-month reperfusion groups in comparison to the control myocardium. Data were analysed by non-paired t-Student’s test. Scoring was performed by a blinded observer unaware of the experimental group. *P < 0.05, **P < 0.01 vs. control. [file 12917_2020_2465_MOESM2_ESM.tiff]

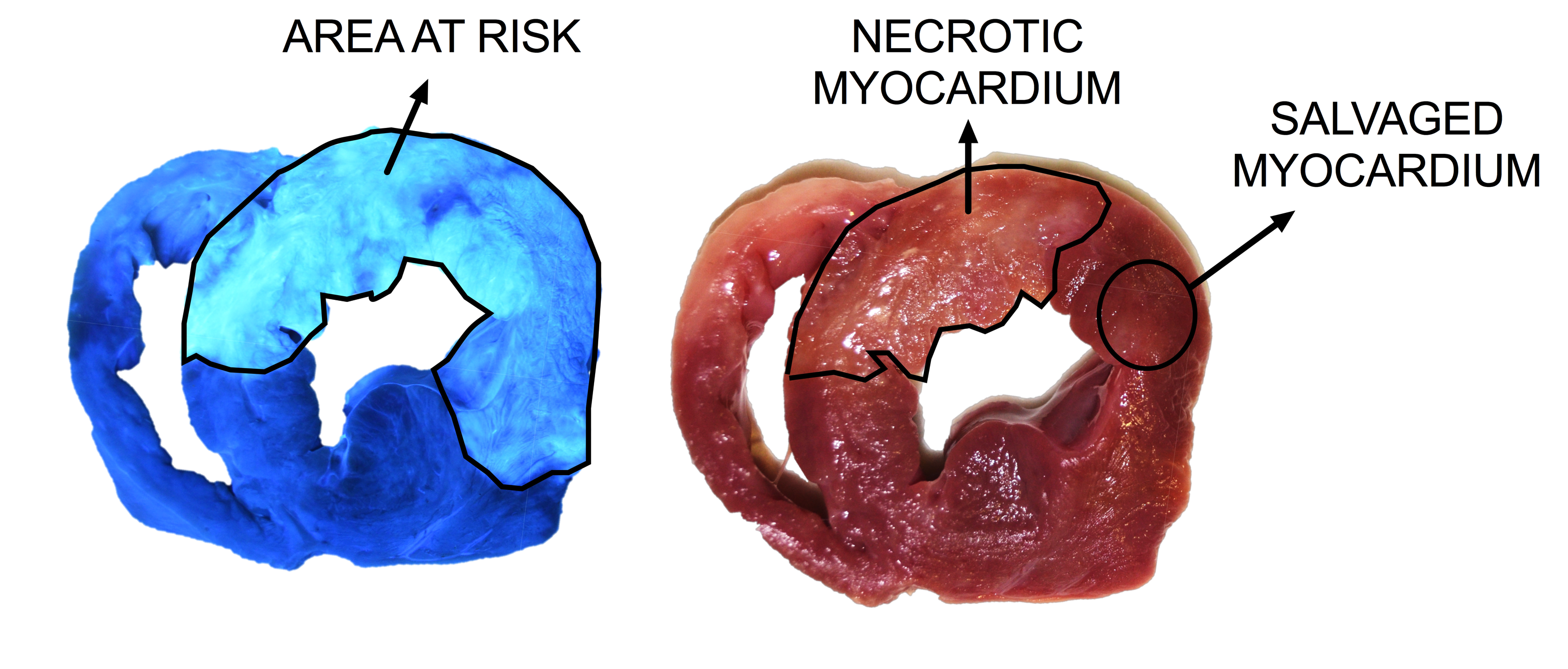

Supplement: Supplementary file 3 — Additional file 3: Supplementary Figure 3. Macroscopic study of myocardial hearts obtained from the swine model. Samples were stained with thioflavin-S (T-S, left) and 2,3,5-triphenyltetrazolium chloride (TTZ, right). Necrotic tissue was defined as the myocardial area stained with TTZ. Salvaged myocardium was defined as the non-infarcted territory within the area at risk clearly outside the infarcted area (with TTZ and T-S staining). [file 12917_2020_2465_MOESM3_ESM.tiff]
